# Commentary: Flow-injection analysis—an idea incomplete?

**DOI:** 10.1155/S1463924682000285

**Published:** 1982

**Authors:** H. W. Holy

**Affiliations:** Technicon International Division S.A. 5 rue Pedro-Meylan Case Postale 64 Geneva 1211 Switzerland


### Commentary: Flow-injection analysis—an idea incomplete?*

**H. W. Holy**

*Technicon International Division S.A., 5 rue Pedro-Meylan, Case Postale 64, 1211 Geneva, Switzerland*

The ultimate aim of any instrumentation must be 'sample in—result out in 1–2 min.'. This specification has been the reason for the phenomenal success of flame analysis, it is the reason for the now remarkable success of the near infra-red analysis for protein, fat etc. and for the continuing research after more than 60 years into all methods of electrode measurements.

Yet Flow-Injection Analysis (FIA) which has often been publicized in the same terms, has still to make a significant impact in the analytical laboratory if by 'impact' we mean systems sold in comparison with the number of Technicon AutoAnalyzers or similar continuous flow systems. A conservative estimate suggests that more than 50 000 continuous flow units are now in the field after about 25 years.

A fully automated FIA-type system was, after all, commercially available in 1959 and described in great detail by Jonnard [1] for the analysis of chromate, protein, urinary glucose, red blood cells and haemoglobin. The fundamental theory of FIA, that is the dispersion of a sample slug in a flowing stream, was first developed by Taylor [2] in 1953 and subsequently by Aris [3] in 1956. The concept was reintroduced into electrochemistry by Pungor's school in 1970 and, since 1976, widely expanded by Ruzicka and his colleagues in the academic press. Today at least four firms market FIA systems actively in Europe and the USA. Hence the very limited acceptance of FIA by practising analysts cannot be blamed on a lack of history, academic support or commercial exploiters.

The goal of FIA is certainly achievable—theoretically. Given completely turbulent flow (Reynold's number greater than 1000), a sample injected into such a stream would be recorded as a complete square wave on any detector. Samples can be injected at hundreds/h, each peak could be monitored for quality of the assay and so duplicates would be unnecessary. Micro samples could be used with high precision and if the reactions were relatively simple, increased reaction rates would often be possible using high temperatures with the high pressures necessary. Separations using packed columns would open new possibilities without significant loss of the square wave. The price? A high/very-high pressure system of narrow bore tubes (0·2 mm?) with velocities in the order of 500 cm/s and possible (but not certainly) high reagent consumption. To my knowledge, the obvious technological problems have never been solved on a commercial instrument.

In contrast, FIA uses conventional low-velocity flow systems. Separation techniques, such as dialysis or solvent extraction techniques on line, have been described but at low analysis rates. Calibration curves are rarely linear, peaks are spiked and so tell nothing of the reliability of each assay. Duplicate and triplicate assays are usually recommended, a feature which unfortunately makes the system very work intensive and hence error prone if a many-sample series is run. The oft-quoted appeal of FIA is instrumental simplicity and hence, by implication, instrument reliability. However, one suspects that FIA has tried to buy its advantages at too cheap a technological price. Certainly new equipment has now addressed the problem with automated diluters as accessories, automated samplers and computerized peak picking and data handling, but instrumental simplicity has now been lost. What has been gained? Does FIA really offer advantages to the practising analyst over conventional methods? How many of the methods published really differ from preparing a solution and subsequently measuring it in a spectrophotometer equipped with a flow-through cuvette, or, at the other extreme, is there really any advantage to performing separations and massive dilutions manually when automated equipment exists? Is there really an advantage, for example, in analysing metals by FIA rather than by atomic absorption? Is there really any advantage in using FIA for acid-based titration in preference to the many excellent acid-based titrators currently on the market? Can it be that the promoters of FIA have not really thought through the problem of exactly where FIA offeres a unique advantage and have dissipated their efforts in 'me too-ism', the 'what you can do we can do as well—well, almost' syndrome?

The answer will come at that time when more of the proponents of FIA take a cold, hard, long look at the technique and answer the questions most analysts would ask. System stability, real throughput rates which includes preparation, equilibrating, calibrating, data interpretation and reasonable operating speeds, real sample volumes which include washing out injection pipettes and valves etc.

Until such time, the real position of FIA in the analyst's repertoire of methods will remain unclear and the number of disillusioned users may well condemn the technology to oblivion.

**Commentaries in the* Journal *express their authors' views only and it should not be considered that the Editor and his advisers necessarily agree with them.*

### References

1. JONNARD, R. *Annals of the New York Academy of Science* (1959), 672–728.

2. TAYLOR, G. *Proceedings of the Royal Society of London*, Series A, **219** (1953), 186.

3. ARIS, R. *Proceedings of the Royal Society of London*, Series A, **325** (1956), 67.

0142–0453/82/0403 0111 $05·00 © 1982 Taylor & Francis Ltd

